# Management of Cervical Spinal Cord Injury without Major Bone Injury in Adults

**DOI:** 10.3390/jcm12216795

**Published:** 2023-10-27

**Authors:** Hideaki Nakajima, Kazuya Honjoh, Shuji Watanabe, Ai Takahashi, Arisa Kubota, Akihiko Matsumine

**Affiliations:** Department of Orthopaedics and Rehabilitation Medicine, University of Fukui Faculty of Medical Sciences, 23-3 Matsuoka Shimoaizuki, Eiheiji-cho, Yoshida-gun, Fukui 910-1193, Japan; kazuya@u-fukui.ac.jp (K.H.); shujiw@u-fukui.ac.jp (S.W.); aitun@u-fukui.ac.jp (A.T.); akubota@u-fukui.ac.jp (A.K.); matsumin@u-fukui.ac.jp (A.M.)

**Keywords:** cervical spinal cord injury, no major bone injury, management

## Abstract

The incidence of cervical spinal cord injury (CSCI) without major bone injury is increasing, possibly because older people typically have pre-existing cervical spinal canal stenosis. The demographics, neurological injury, treatment, and prognosis of this type of CSCI differ from those of CSCI with bone or central cord injury. Spine surgeons worldwide are debating on the optimal management of CSCI without major bone injury. Therefore, this narrative review aimed to address unresolved clinical questions related to CSCI without major bone injury and discuss treatment strategies based on current findings. The greatest divide among spine surgeons worldwide hinges on whether surgery is necessary for patients with CSCI without major bone injury. Certain studies have recommended early surgery within 24 h after injury; however, evidence regarding its superiority over conservative treatment remains limited. Delayed MRI may be beneficial; nevertheless, reliable factors and imaging findings that predict functional prognosis during the acute phase and ascertain the necessity of surgery should be identified to determine whether surgery/early surgery is better than conservative therapy/delayed surgery. Quality-of-life assessments, including neuropathic pain, spasticity, manual dexterity, and motor function, should be performed to examine the superiority of surgery/early surgery to conservative therapy/delayed surgery.

## 1. Introduction

Spinal cord injury (SCI) can have severe consequences, leading to a loss of neurological function, which can significantly impact a patient’s quality of life (QOL) and cause significant economic harm. A recent review found the incidence of SCI to range from 5.1 to 150.48 cases per million based on national studies undertaken between 2013 and 2020 [1]. Annual national incidence rates range from 8 to 50 per million depending on the country [2,3]. Notably, the characteristics of SCI have undergone progressive changes. Over the past four decades, the average patient age has increased, while the proportion of those with no fractures, incomplete injuries, or partial injuries only has risen. Conversely, the number of patients with complete injuries has decreased [4]. A nationwide survey in Japan revealed that SCI is most prevalent among patients in their 70s, with cervical SCI (CSCI) without major bone injury accounting for 70.7% of all CSCI cases resulting from minimal trauma [5].

According to the “time is spine” concept, it is globally accepted that surgical treatment should be performed within 24 h in patients with SCI and concurrent bone injury, such as dislocation fracture, as this augments its beneficial impact on functional and neurological improvement [6]. However, a significant disagreement exists among spine surgeons globally regarding the management of CSCI without major bone injury in terms of “opting between conservative and surgical treatment”, “surgical timing”, “the availability of magnetic resonance imaging (MRI)”, and “prognostic factors”, despite the fact that it is the most prevalent form of incomplete CSCI in the aging society. In this regard, the number of articles on the management of patients with CSCI without major bone injury has increased, especially in recent years. Recent interactive discussions among experts from the AOSpine Spinal Cord Injury Knowledge Forum proposed that neurological status, spinal instability, and the presence of spinal cord compression were the most significant variables influencing spine surgeons’ decision making regarding the role and timing of surgery [7]. However, no highly evidence-based consensus currently exists regarding the optimal management plan for CSCI without major bone injury.

Therefore, this review aimed to address unresolved clinical questions related to CSCI without major bone injury and discuss the strategies for treating affected patients based on current findings. A narrative review was conducted by searching PubMed from January 2010 to July 2023 using the following search strategy: “cervical spinal cord injury” and “without bone injury”; “without fracture”; and “without radiological abnormality”. After the preliminary screening of the titles and abstracts of each article, full-text assessments were performed. This review included prospective studies, retrospective studies (>30 cases), review articles, and important articles relevant to cervical spinal cord injury without major bone injury.

## 2. Relationship between Cervical Spinal Canal Stenosis (CSCS) and the Incidence of CSCI without Major Bone Injury

### 2.1. Incidence of CSCS in Patients with CSCI without Major Bone Injury

CSCS can be caused by various pathological factors, including ossification of the posterior longitudinal ligament (OPLL), developmental/congenital narrow canal (a spinal canal diameter of <12–13 mm in classical diagnostic criteria), vertebral osteophyte, bulging of the intervertebral disc, and calcification of the yellow ligament [8,9]. These pre-existing factors potentially increase the risk of CSCI without major bone injury. In previous studies, 86% [10] and 74% [11] of patients had CSCS. Furthermore, studies in Japan found the prevalence of OPLL to be 39% [12], 34% [13], 35% [10], and 34.3% [14] in patients with CSCI without major bone injury. A previous study reported the likelihood of developing CSCI to be 124.5 times greater in individuals with CSCS compared with that in those without [15]. Considering the higher prevalence of pre-existing CSCS and incidence of CSCI without major bone injury in Japan than in European countries [16], pre-existing CSCI is undoubtedly a potential risk factor for CSCI without major bone injury. A review of MRI measurements showed that cord–canal mismatch (a higher spinal cord occupation ratio in the spinal canal) was more important and should be viewed as the underlying mechanism predisposing individuals to SCI [17]. In addition, a report suggests that improving the cervical curvature can positively impact prognosis, as the cervical curvature in patients with total spinal balance can be affected by nonsurgical treatment for CSCI without major bone injury [18].

### 2.2. Association of CSCS with Neurological Severity and the Need for Prophylactic Surgery

Several reports have been skeptical regarding the relationship between the presence of pre-existing CSCS and the severity of neurological symptoms associated with injury [12,19,20]. A previous report claims that the development of traumatic CSCI can be prevented in only 0.017% of individuals with CSCS if they undergo decompression surgery before experiencing trauma [15]. In a prospective study of patients with cervical OPLL, only 2% of patients without myelopathy at the time of the initial consultation subsequently developed trauma-induced myelopathy. In contrast, 13% of patients with myelopathy identified cervical trauma as the trigger of their myelopathy [21]. Another multicenter, retrospective study suggested that 29% of patients experienced worsening subjective symptoms owing to falls. However, a preoperative Japanese Orthopaedic Association (JOA) score cut-off point of eight was determined to predict a high risk of worsening motor impairment, potentially implying the presence of severe myelopathy even before the fall [22]. Previous studies have suggested that preventive surgical management for patients with CSCS may be unnecessary if they present no neurological symptoms, although it is important to inform patients that CSCS is a risk factor for CSCI [19].

## 3. Clinical Significance of MRI

### 3.1. Relationship between an Increased Spinal Cord Signal Intensity and Neurological Prognosis

Most patients with CSCI without major bone injury exhibit increased signal intensity (ISI) in MRI, and certain studies have reported a relationship between ISI in MRI and neurological outcomes [23]. In a multicenter study in Japan, ISI had a negative impact on neurological improvement in American Spinal Injury Association Impairment Scale (AIS) A–C cases [14]. A systematic review of MRI findings, including those of patients with SCI with or without major bone injury, reported that longer intramedullary hemorrhage, a smaller canal diameter at maximal spinal cord compression, and the presence of spinal cord edema were found to be associated with poor neurological recovery [24]. ISI changes in the spinal cord potentially reflect initial spinal cord injury, and some reports have suggested that the grade (none, light, and intense) and range of ISI are correlated with neurological severity at injury [25,26,27].

### 3.2. Association of Paraspinal Soft Tissue and Ligament Damage with Neurological Severity and Outcome

The degree of cervical paraspinal soft tissue damage after cervical SCI without major bone injury is also an important MRI finding regarding the relationship between the degree of the prevertebral high-intensity area (HIA) and severity of neurological symptoms at injury and outcomes [28,29]. A report suggests that posterior paraspinal soft tissue damage exceeding the nuchal ligament within 24 h after injury is significantly related to neurological improvement but not to anterior damage [30]. Regarding the pathomechanisms underlying cervical SCI without major bone injury, severe hyperextension injury has been found to cause tensile stress in the anterior column and compressive stress in the posterior column. The presence of an HIA exceeding the nuchal ligament potentially suggests greater energy at injury [31,32].

### 3.3. Association between the Degree of Spinal Cord Compression and Neurological Prognosis

The association between the degree of cervical spinal cord compression and neurological symptoms in patients with SCI without major bone injury is also controversial. Based on expert panel discussions and international surveys, the presence of spinal cord compression is an important variable influencing the management of acute SCI [7]. A retrospective study demonstrated that severe cervical spinal cord compression of >40% had a significant impact on the level of paralysis at the time of injury [33]. Another study revealed that individuals with spinal cord compression exceeding 33.2% who underwent surgery demonstrated a significantly greater chance of walking independently at follow-up than those who received conservative treatment [34]. However, several studies have established no connection between CSCS and neurological outcomes [20,28,30].

### 3.4. Clinical Significance of Delayed MRI Compared with That of Acute MRI

An additional important finding discovered by recent studies regarding the clinical significance of MRI findings is that delayed MRI (after 2 weeks) more accurately reflects clinical symptom severity than acute MRI (within 2 days) because of the contrasting ISI and paraspinal HIA findings according to MRI timing [35,36]. A study reported that the degree of the paraspinal HIA and range of ISI in the spinal cord were decreased with delayed MRI, which displayed a strongly negative correlation between the intensity of ISI and the JOA score; nonetheless, this correlation was not present for acute MRI. As the disease progresses, the ISI can range from none to mild ISI, and subsequently to intense ISI, eventually leading to a decreased post-surgical recovery rate [35]. In particular, ISI tends to be worse in patients with SCI without major bone injury than in those with cervical spondylotic myelopathy after surgical treatment [37]. Delayed and careful follow-up MRI may be recommended regardless of the treatment strategy [38].

## 4. Opting between Conservative and Surgical Treatment and Determining the Optimal Timing for the Treatment of CSCI without Major Bone Injury

The most crucial clinical question, which is causing the greatest division of opinion among spinal surgeons worldwide, regarding the optimal management plan for CSCI without major bone injury is the lack of high evidence-based studies. While patients with CSCI without major bone injury are anticipated to experience some spontaneous neurological improvement without undergoing surgical decompression, predicting the prognosis of neurological status in the acute phase remains difficult.

### 4.1. Comparison between Conservative and Surgical Treatment

Two prospective studies in Japan found that surgery was not more effective than conservative treatment for patients with CSCI without major bone injury in terms of improving paralysis [39,40]. While these prospective studies’ results are critical, limitations persist in interpreting the results in terms of the number of cases, degree of spinal cord compression, and surgical timing. A multicenter, retrospective study reported no significant differences between the surgical and conservative treatment groups in terms of the ASIA impairment scale grade, ASIA motor score 6 months after injury, and change in the ASIA motor score from baseline to 6 months after injury, regardless of surgical timing [41,42]. In contrast, a retrospective study suggested that surgical treatment should be recommended to restore walking ability in patients with spinal cord compression of ≥33.2% and who cannot remain in a seated position without assistance during the post-injury subacute phase, although conservative treatment is basically recommended [34].

### 4.2. Efficacy of Early Surgery Compared with That of Delayed Surgery in Improving Neurological Outcome

In the debate over the superiority of surgery versus conservative treatment, the efficacy of early surgery (within 24 h after injury) should be discussed. A pooled analysis derived from multicenter data sources suggested that surgical decompression within 24 h of acute SCI is associated with improved sensorimotor recovery [43]. A retrospective analysis of observational data from North America found that patients with SCI who underwent early surgery without instability experienced significantly greater motor recovery, with an additional 6.31 points on the Motor Recovery Scale; 2.81 times greater odds of converting to a different AIS grade at 12 months; and an additional 7 points on the Functional Independence Measure at 6 months after injury compared with those who underwent late surgery, although no significant differences in AIS at follow-up were noted [44]. A retrospective, comparative study reported that early surgical treatment resulted in better neurological outcomes than conservative treatment in patients with incomplete CSCI without major bone injury and suggested that it may prevent secondary deterioration in these patients following the initial insult [45]. An AO Spine-led multidisciplinary guideline development group recommended weakly early decompression for patients with acute central cord syndrome, considering the potential benefits for neurological and functional outcomes [46]. However, in a prospective study of AIS C patients with CSCI without major bone injury, early surgical treatment (<24 h) yielded similar motor restoration 1 year after injury to delayed surgical treatment (>2 weeks) [47].

### 4.3. Importance of QOL and Motor Function Assessments

Although motor function improvement tends to receive the most attention when discussing treatment strategies, improvements in QOL, such as the alleviation of chronic stiffness and neuropathic pain, may be of particular interest in patients with CSCI without major bone injury. In a cross-sectional study, the incidence of NeP was 65% [48]. Previous studies have indicated that 37–50% of these patients experience pain at the spinal segment level, while 76–83% experience pain below the level of the lesion [49,50]. It may be necessary to avoid concluding that surgical treatment is not more significant than conservative treatment in patients with CSCI without major bone injury based on the finding wherein no significant difference in motor function recovery was observed.

## 5. Surgical Procedure: Anterior vs. Posterior Approach

### 5.1. Presence of Disc Rupture and Surgical Approach

Anterior surgical decompression can alleviate instability caused by ligament complex injuries; however, it often requires the fusion of multiple vertebral segments, a process that potentially results in the loss of motor function. Posterior laminoplasty provides a broader range of decompression than anterior surgery and can preserve motion segments; nevertheless, it may lead to cervical instability in patients with segmental instability.

The posterior approach is potentially more advantageous for multilevel CSCS; nonetheless, most patients with CSCI without major bone injury have disc rupture. The risk of aggravating an existing spinal cord injury or developing chronic neck pain and deformity increases owing to disc-rupture-induced instability [51]. For these cases, spinal fixation (anterior approach) may be feasible; however, no consistent diagnostic method is available for determining whether a patient has cervical disc rupture. A typical feature of cervical disc rupture is a high disc signal and ALL discontinuity in MRI at injury [52]. However, certain reports have indicated that numerous patients with CSCI without major bone injury having concomitant disc injury tend to be overlooked owing to a lack of typical MRI features [28,53]. A previous study reported that prevertebral hematoma, high-signal SCI, and a high-signal posterior ligament complex (PLC) were valuable in diagnosing traumatic cervical disc rupture, with a high-signal PLC being particularly noteworthy [32].

### 5.2. The Clinical Differences between Anterior and Posterior Surgery

A retrospective, comparative study found no significant differences between anterior and posterior surgery in multi-level CSCS patients with CSCI without major bone injury [54]. Another study reported the clinical significance of anterior fusion surgery but suggested that three-segment anterior fusion surgery is not superior to that of short-segment fusion (single- or double-segment) for CSCI without major bone injury [55]. Differences in the possible complication profiles based on the surgical approach are shown. The anterior approach carries the risk of developing dyspnea, dysphagia, dislodgement and subsidence of the bone graft or cage, and adjacent segment disorder. This risk increases with an increase in the number of fused segments. The posterior approach also carries the risk of postoperative kyphotic deformity, axial neck pain, and wound infection [54,56].

## 6. Management of Conservative Treatment and Rehabilitation

### 6.1. Necessity of Collar Immobilization in the Acute Phase: Rigid- or Soft-Collar Fixation

Immobilization of the cervical spine using a rigid collar is recommended for patients with cervical spine injuries to prevent further injury, according to both the Advanced Trauma Life Support guidelines of the American College of Surgeons [57] and the Prehospital Trauma Life Support guidelines of the National Association of Emergency Medical Technicians [58]. Other recent protocols, such as those of the Queensland Ambulance Service, South Eastern Sydney Local Health District, Illawarra Shoalhaven Local Health Districts, and Victorian Ambulance Services, with the support of the Australian and New Zealand Committee on Resuscitation, have been revised to solely recommend soft-collar immobilization in the prehospital setting for potential cervical spine injuries [59,60,61,62]. A systematic review and meta-analysis found that rigid collars provide significantly better stability than no or soft collars in flexion/extension and rotation movements. However, clinical studies have not confirmed any differences in neurological outcomes. The review concluded that no clinical evidence is available to support the need to apply a rigid collar, and soft collars provide adequate stability and a sense of immobilization for patients, leading to increased compliance [63].

### 6.2. Clinical Benefit of Rehabilitation

Functional recovery in patients cannot be achieved without rehabilitative intervention. In addition to conventional rehabilitation, the usefulness of recently developed electrical stimulation therapy and robotic-assisted physiotherapy has also been reported [64,65]. A meta-analysis revealed that transcranial magnetic stimulation led to improvements in walking speed and lower-extremity function. Similarly, functional electrical stimulation significantly increased upper-extremity independence. Additionally, robotic-assisted treadmill training was found to enhance lower-extremity function compared with related controls [66].

In patients with CSCI without major bone injury, the management of respiratory dysfunction is extremely important because CSCI that affects the C3–4 level is likely to cause damage to the spinal respiratory center, including the phrenic nerve [67]. A previous study reported that restrictive ventilatory impairment was present in 92.6% of patients with CSCI without major bone injury upon initial evaluation. Respiratory rehabilitation, such as expiratory-muscle resistance training, should be continued for ≥12 weeks after CSCI without major bone injury. An improvement in lung capacity has been observed as the lower limbs regain their motor function [68].

### 6.3. Neuropathic Pain after Spinal Cord Injury

Pain affects a large proportion of individuals with SCI, with 65–85% of patients with SCI experiencing pain. Of these, one-third describe the pain as severe and persistent, often worsening over time, especially if it occurs within 6 months of the injury [69]. Pain has a significant impact on patients’ activity levels and mental health and negatively affects their QOL [70]. The most common type of post-SCI neuropathic pain is paresthesia/dysesthesia, and patients with AIS B have reported more intense neuropathic pain than those with other grades [50]. A randomized, double-blind, placebo-controlled, phase 3 study found mirogabalin to be a promising treatment for patients with SCI-induced neuropathic pain [71]. Another study suggested that mirogabalin was the most effective treatment for paresthesia/dysesthesia; nevertheless, regarding its treatment efficacy, paresthesia/dysesthesia was the most difficult to relieve pain compared with other types of neurological pain [72].

## 7. Prognostic Factors

In a study that identified the highest number of prognostic factors for CSCI without major bone injury, body mass index, spinal cord signal intensity in MRI, AIS on admission, comorbid dementia/delirium, and post-injury pneumonia were found to be independently and significantly associated with walking recovery in patients with AIS A–C. Additionally, the prevalence of OPLL was found to be an independent prognostic factor for AIS B–C cases [14]. The presence of comorbid conditions, such as dementia/delirium and post-injury pneumonia, can hinder the prospects of rehabilitation, leading to poorer neurological improvement. Other studies have suggested that younger age, cervical myelopathy at baseline, spinal cord compression ratio, and time from injury to treatment are independent prognostic factors [73,74,75]. Previous studies have demonstrated that physical examinations are a reliable method for predicting neurological improvement in patients with non-surgically treated C3–4 CSCI without major bone injury. These studies’ participants were found to have a positive prognosis for upper-extremity function when they were able to perform hip and knee flexion from the supine position and elbow extension 3 days after injury [76].

## 8. Discussion

Recent epidemiological studies from Europe, East Asia, and North America have consistently demonstrated that motor-incomplete injuries (AIS C and D), particularly those without major bone injury, are on the rise, a trend that may be linked to the aging of the global population [4,5,77].

A prospective cohort study of patients with central cord injury (weakness in the upper extremities and less severe weakness in the lower extremities) from the Canadian national SCI registry reported that CSCI patients with stable spines were older, presented with more medical comorbidities, had injuries at higher cervical levels (C1–4), experienced less severe neurological injuries (ASIA C or D), and had greater total motor score changes than those with unstable spines [78]. The clinical relevance of managing patients with CSCI without major bone injury is greater than that of managing those with classic central cord injury, as this subpopulation has been discovered to be distinct regarding demographics, neurological injury, treatment, and prognosis. A significant proportion of patients with CSCI without major bone injury constitute subgroups that should be further categorized to determine the most effective treatment strategy in cases where it is uncertain whether surgery should be performed. In a multicenter study in Japan, the prevalence of central cord syndrome was 38.6% and 85.4% in AIS C and D cases, respectively.

This review focused on the management of patients with CSCI without major bone injury. The most controversial issue in these patients is the need for surgery. The management of CSCI without major bone injury remains largely based on individual decisions, despite the lack of convincing evidence for either surgical or conservative treatment. Although several recent studies have recommended early surgery within 24 h after injury, considerably few prospective studies have investigated this patient group in this regard. Furthermore, it should not be ignored that among these few prospective studies, none have demonstrated the superiority of surgery over conservative therapy [39,40] (Table 1). Numerous studies have suggested that early surgery is superior to delayed surgery. Even in these prospective studies, a conclusion regarding surgical treatment could not be drawn because most of the surgically treated cases in these studies were in the delayed surgery group. Moreover, a prospective study on AIS C patients with CSCI without major bone injury also reported that the superiority of early surgical treatment (<24 h) for motor recovery to delayed surgical treatment (>2 weeks) at 1 year after injury could not be confirmed [47] (Table 1). Although early surgery is the preferred option when a patient is selected for surgical treatment, according to previous reports, a more important issue is that these patients often achieve improvement in neurological function during the natural post-injury course; furthermore, no established method is available to accurately predict the degree of subsequent recovery during the acute phase. It should be noted that some of the patients included in the studies who apparently exhibited the effectiveness of early surgery could have sufficiently improved even without surgery. In contrast, many spine surgeons have witnessed improved motor function immediately after decompression surgery, even where patients did not show any signs of recovery for several weeks leading up to surgery. This controversy suggests that surgical treatment may be effective for patients with CSCI without major bone injury, and such surgery can result in significant neurological improvement. In addition, assessing QOL, such as spasticity, pain, manual dexterity, and motor function, especially in patients with CSCI without major bone injury, may be a prerequisite to discussing the superiority of surgery/early surgery over conservative therapy/delayed surgery. If surgical treatment leads to improved QOL, even without prioritizing motor function recovery, it should be considered.

In this context, formulating a method that can identify patients with better prospects of neurological recovery if surgery is performed earlier in the post-injury period is desirable. The clinical significance of delayed MRI (>2 weeks after injury) was suggested in a prospective study; however, determining the need for surgery and implementing it as soon as possible are both difficult tasks. Other objective assessments, such as machine learning [79,80,81], biomarkers [82,83,84], and novel imaging technologies [85,86,87], are required to evaluate neurological prognoses and treatment strategies.

## 9. Conclusions

CSCI without major bone injury is the most common type of SCI in the worldwide aging society. Early surgery within 24 h after injury has been increasingly recommended in recent years; however, no prospective study has demonstrated the superiority of surgery over conservative therapy or early surgery over delayed surgery for patients with CSCI without major bone injury. In addition, evaluating the need for surgery in these patients during the acute phase is difficult because most of them have the potential to recover naturally. However, previous studies have indicated that some patients are likely to experience better functional recovery if surgery is performed. Establishing evaluation methods that can predict functional prognosis at an earlier stage and enable the timely selection of treatment strategies is warranted. In addition to motor recovery, future research should also include other measures relevant to management and outcomes such as the assessment of QOL, including neuropathic pain, manual dexterity, and adverse events.

## Figures and Tables

**Table 1:** Prospective studies on patients with cervical spinal cord injury without major bone injury.

	Ref.	Inclusion Criteria	Number of Patients	Main Results
Conservative vs. surgical treatment	[35]	AIS B and C Spinal cord compression rate > 20%	Surgery (n = 17) Conservative (n = 17)	The two groups exhibited almost the same course in their recovery processes (no significant difference).
	[36]	AIS A, B, and C Spinal cord compression rate > 20%	Surgery (n = 11) Conservative (n = 11)	No significant difference in paralysis improvement was observed. A higher frequency of surgical complications was noted.
Early vs. delayed surgery	[42]	20–79 years Pre-existing canal stenosis AIS C	Early (n = 36) Delayed (n = 36)	Early surgical treatment produced similar motor restoration 1 year after injury to delayed surgical treatment.
Significance of MRI findings	[32]	Diagnosed within 48 h 40 patients underwent surgery after delayed MRI	n = 68	The increased signal intensity grade in delayed MRI displayed a significantly negative correlation with the JOA score; however, this correlation was absent in acute MRI.
	[24]	Consecutive patients Underwent expansive laminoplasty	n = 36	Preoperative grayscale of signal intensity and range of signal intensity were predictors of surgical outcomes.

## Data Availability

No new data were created.
